# Knock-Down of a Tonoplast Localized Low-Affinity Nitrate Transporter *OsNPF7.2* Affects Rice Growth under High Nitrate Supply

**DOI:** 10.3389/fpls.2016.01529

**Published:** 2016-10-25

**Authors:** Rui Hu, Diyang Qiu, Yi Chen, Anthony J. Miller, Xiaorong Fan, Xiaoping Pan, Mingyong Zhang

**Affiliations:** ^1^Key Laboratory of South China Agricultural Plant Molecular Analysis and Genetic Improvement and Guangdong Provincial Key Laboratory of Applied Botany, South China Botanical Garden, Chinese Academy of SciencesGuangzhou, China; ^2^University of Chinese Academy of SciencesBeijing, China; ^3^Metabolic Biology Department, John Innes CentreNorwich, UK; ^4^State Key Laboratory of Crop Genetics and Germplasm Enhancement, Nanjing Agricultural UniversityNanjing, China

**Keywords:** *OsNPF7.2*, nitrate, transporter, growth, *Oryza sativa*

## Abstract

The large nitrate transporter 1/peptide transporter family (NPF) has been shown to transport diverse substrates, including nitrate, amino acids, peptides, phytohormones, and glucosinolates. However, the rice (*Oryza sativa*) root-specific family member *OsNPF7.2* has not been functionally characterized. Here, our data show that OsNPF7.2 is a tonoplast localized low-affinity nitrate transporter, that affects rice growth under high nitrate supply. Expression analysis showed that *OsNPF7.2* was mainly expressed in the elongation and maturation zones of roots, especially in the root sclerenchyma, cortex and stele. It was also induced by high concentrations of nitrate. Subcellular localization analysis showed that OsNPF7.2 was localized on the tonoplast of large and small vacuoles. Heterologous expression in *Xenopus laevis* oocytes suggested that OsNPF7.2 was a low-affinity nitrate transporter. Knock-down of *OsNPF7.2* retarded rice growth under high concentrations of nitrate. Therefore, we deduce that *OsNPF7.2* plays a role in intracellular allocation of nitrate in roots, and thus influences rice growth under high nitrate supply.

## Introduction

Nitrogen (N) is one of the most important macro elements in plants, essential for growth and development. Most plants need to uptake N through their roots from the soil. In agriculture, crops are generally fertilized with large amounts of N to obtain high yields, even though more than half of the N added to cropland can be lost to the environment (Lassaletta et al., 2014). Therefore, it is important to study the uptake, transport and assimilation of N for effective fertilizer management practices. N as a fertilizer is acquired mainly in the form of ammonium and nitrate by roots (Xu et al., 2012). The nitrate taken up from the rhizosphere is reduced to nitrite in the plant, which is then further reduced to ammonium. Ammonium is then subsequently assimilated to glutamine, and further to glutamic acid and other forms of organic N.

Nitrate and ammonium can be used as the major N sources of rice (Kronzucker et al., 1999). After uptake from the soil, only 37% of incoming nitrate and 24% of incoming ammonium can be translocated to shoot. However, 52–53% of the N absorbed can be assimilated and compartmentalized to the vacuole in rice (Kronzucker et al., 2000). Therefore, the vacuole plays an important role in efficient N utilization in rice.

The nitrate transporter 1/peptide transporter family (NPF, also known as the NRT1 family) is related to the SLC15/PepT/PTR/POT family of peptide transporters in animals (Léran et al., 2014). In plants, the reported members of the NPF can transport not only peptides, but also nitrate, nitrite, amino acids, phytohormones and glucosinolates (Zhou et al., 1998; Tegeder and Rentsch, 2010; Kanno et al., 2012; Nour-Eldin et al., 2012; Léran et al., 2014; Pike et al., 2014). It is worth mentioning here that many members of this family have exhibited nitrate transport activity (Léran et al., 2014).

The mechanism of nitrate transport and function of some NPFs has been investigated in *Arabidopsis*. Unlike the high-affinity nitrate transporter family (NRT2 family), the NRTs of the NPF with the exception of AtNPF6.3, are low-affinity NRTs. Previously known as AtCHL1 or AtNRT1.1, AtNPF6.3 is a dual-affinity NRT (Liu et al., 1999), and was first reported as a nitrate-inducible plant NRT (Tsay et al., 1993). Later, it was ascertained to be a nitrate sensor (Ho et al., 2009), which changed auxin distribution in response to different nitrate conditions to module the root structure (Krouk et al., 2010). It was also found that calcium acted as downstream signal of *AtNPF6.3* (Riveras et al., 2015). The crystal structure of the AtNPF6.3 protein has been described (Parker and Newstead, 2014; Sun et al., 2014). Apart from AtNPF6.3, *Arabidopsis* is also known to have many NPFs nitrate transporter to facilitate a multitude of functions.

Unlike *Arabidopsis*, few rice NPF genes have been investigated. OsNPF8.9 (OsNRT1) was reported as a NRT after expression in *Xenopus* oocytes (Lin et al., 2000). The role of *OsNPF4.1* (*SP1*) has been demonstrated in the rice panicle elongation (Li et al., 2009) and the overexpression of *OsNPF8.20* (*OsPTR9*) improved N utilization efficiency, growth and grain yield (Fang et al., 2013). However, the transported substrates of OsNPF4.1 and OsNPF8.20 remain unknown. OsNPF6.5 (OsNRT1.1B) showed dual-affinity nitrate transport activity, and it diverged between *indica* and *japonica* rice cultivars during evolution. The *OsNPF6.5-indica* variation had enhanced N use efficiency (Hu et al., 2015). In addition, two NRTs, OsNPF2.4 (Xia et al., 2015) and OsNPF2.2 (Li et al., 2015), participated in long distance root-to-shoot nitrate transport. Knockout of *OsNPF2.4* impaired potassium (K)-coupled nitrate upward transport and nitrate-redistribution from old leaves to N-starved roots and young leaves. Moreover, knockout of *OsNPF2.4* increased the shoot: root ratio of tissue K under higher nitrate (Xia et al., 2015).

To secure their N supply, plants have multiple transport systems for N uptake from the soil as well as for intra- and intercellular reallocation of N containing compounds. Vacuole compartmentation is an important part of nitrate utilization at intracellular level. Nitrate is imported into vacuoles under conditions of abundant nitrate outside, and exported to cytosol to meet subsequently nitrate deficiency in the environment. Several fold more nitrate was measured in vacuoles than cytosol (Martinoia et al., 1981; van der Leij et al., 1998). Plants need active transporters to overcome the concentration gradient between vacuoles and cytosol. However, the transporters on the vacuolar membrane for this function are rarely described. A chloride channel (CLC) protein family member AtCLCa was reported as a vacuolar nitrate/proton antiporter in *Arabidopsis* (De Angeli et al., 2006). The NRT2 family member AtNRT2.7 was found to be localized on tonoplast and facilitated nitrate accumulation in the seed (Chopin et al., 2007).

Many NPFs localized on the plasma membrane mediate intercellular allocation of nitrate, but little is known about intracellular nitrate transport. Only a few members of NPF were found to be localized to intracellular membranes. For example, AtPTR2, AtPTR4 and AtPTR6 were localized at the tonoplast (Weichert et al., 2012). AtPTR2 was shown to be a peptide transporter, but the function of *AtPTR4* and *AtPTR6* was not clear. AtNPF3.1, a nitrate/nitrite transporter (Pike et al., 2014) and GA influx carrier cross cell membranes, was localized at the plasma membrane and displayed intracellular membrane compartment localization (Tal et al., 2016). The cucumber nitrite transporter CsNPF3.2 (CsNitr1-L) was localized on the chloroplast (Sugiura et al., 2007). Here, we characterized a tonoplast localized member of the rice NPF family.

On analysis of a public expression database RiceXPro^1^, *OsNPF7.2* was found to be mainly expressed in roots, this was verified by our qPCR and GUS staining of promoter-GUS transgenic rice. Heterologous expression in *Xenopus laevis* oocytes suggested that OsNPF7.2 is a low-affinity NRT. OsNPF7.2 was localized on the membrane of large and small vacuoles. Knock-down of *OsNPF7.2* caused rice growth retardation under high nitrate supply. Our results suggest OsNPF7.2 plays an important role in nitrate accumulation and homeostasis in rice.

## Materials and Methods

### Plant Materials and Growth Conditions

The rice cultivar used in this study was the *japonica* rice variety Zhonghua 11 (ZH11), except for the special annotation. The hydroponic experiments were conducted using the modified rice nutrient solution of the International Rice Research Institute (IRRI solution contains 1.43 mM NH_4_NO_3_, 0.32 mM NaH_2_PO_4_, 0.51 mM K_2_SO_4_, 1 mM CaCl_2_, 1.65 mM MgSO_4_, 8.9 μM MnSO_4_, 0.5 μM Na_2_MoO_4_, 18.4 μM H_3_BO_3_, 0.14 μM ZnSO_4_, 0.16 μM CuSO_4_, 40 μM FeSO_4_) at ambient conditions of 28°C, 14 h light, 10 h dark (Yoshida et al., 1976). For growth in 1/2 MS (Murashige and Skoog, 1962) medium, seeds were sterilized with 5% sodium hypochlorite solution then washed with water. For the various treatments, the N source of the IRRI solution or 1/2 MS was changed.

For short-term induction experiments, ZH11 plants were germinated in sterile conditions, and then grown on the IRRI solution for 2 weeks. Before treatment, the plants were transferred for a 3-day nitrate-starvation, in which (NH_4_)_2_SO_4_ served as sole N source, and then placed in the IRRI solution substituted with high and low concentrations of KNO_3_ as the N supply. The IRRI solution containing KCl (no N) was used as control. For long-term expression analysis, plants were grown on 1/2 MS medium containing different concentrations of KNO_3_ for 2 weeks. In the long-term experiment, ammonium was used to maintain the total N concentrations equal in the medium.

### Real-Time PCR

Total RNA was isolated using RNAiso Plus following the manufacturer’s instructions (Takara, Japan). The synthesis of cDNA was carried out using the Takara Reverse Transcriptase M-MLV (RNase H-) (Takara, Japan). The qPCR was performed to monitor gene expression, and *UBC* (LOC_Os02g42314.2) (Jain et al., 2006) was used as the reference. The procedure (qPCR) was carried out in the presence of the double-strand DNA-specific dye SYBR Green I (SYBR^®^ Premix Ex Taq GC Takara, Japan) and monitored in real time with the Roche LightCycler 480 system (Roche, Switzerland). Semi-quantitative PCR was implemented using *OseEF-1α* (LOC_Os03g08010.1, or LOC_Os03g08020.1) as reference.

### Vectors Construct

For *P_OsNPF7.2_: GUS* construction, a 1576-bp promoter fragment containing the 5′ UTR of *OsNPF7.2* was amplified by PCR. Then this fragment was inserted into the clone vector *pGEM-T Easy* (Promega, China). Subsequently, the fragment was sequenced at Invitrogen (China). The sequenced fragment was then inserted into *pCambia1301* to replace the *35S* promoter via *Sac*I and *Nco*I. Primers are listed in **Supplementary Table S1**. For RNAi construction, the vector *pTCK303* (Wang et al., 2004) was used. To avoid disturbing other homologous genes, 122-bp 5′ UTR of *OsNPF7.2* was used as the *OsNPF7.2-RNAi* fragment. The 122-bp fragment in 5′ UTR of *OsNPF7.2* was cloned to *pTCK303* by *Bam*HI and *Kpn*I for the sense strand, and *Spe*I and *Sac*I for the antisense strand.

### Transformation of Rice

The constructs were introduced into the *Agrobacterium tumefaciens* strain EHA105. Then the *japonica* rice (*Oryza sativa* L.) variety ZH11 was transformed with the *Agrobacterium*-mediated transformation method as previously described (Hiei et al., 1997).

### Subcellular Localization

For *35S: OsNPF7.2: EGFP* construction, the *EGFP* was introduced into the BiFC vector *pSAT1A-nEYFP-N1* (Li et al., 2014) to replace *nEYFP* via *Xba*I and *Kpn*I, then the CDS without stop codon of *OsNPF7.2* was cloned into the vector via *Xho*I and *Eco*RI. Moreover, a linker (GGGS)_2_ was inserted between the CDS of *OsNPF7.2* and *EGFP*. Rice protoplasts were isolated and transformed by using a previously described protocol (Zhang et al., 2011). The transformed protoplasts were observed with confocal laser scanning microscope (Leica TCS SP5, Germany) with 488 nm exciting wavelength for GFP and 543 nm exciting wavelength for mCherry. The images were coded green for GFP and red for mCherry.

### Histochemical Analysis and Section

The construct *P_OsNPF7.2_: GUS* was transformed into ZH11. The transgenic *P_OsNPF7.2_: GUS* rice seeds were sown on 1/2 MS medium. The GUS stain was performed for 4 h except special annotation with 0.5 mg mL^-1^ X-Gluc (5-bromo-4-chloro-3-indolyl-β-D-glucuronide), 3 mM K_4_Fe(CN)_6_, 3 mM K_3_Fe(CN)_6_, 0.05% Triton X-100, and 100 mM Na_2_HPO_4_-NaH_2_PO_4_, pH 7.0. The reaction was stopped by 70% ethanol. After staining, tissues were fixed in a glutaraldehyde solution at 4°C, and embedded in Spurr’s resin. The samples were sectioned into 3 μm thickness and observed under the microscope (ZISS AXOPLAN2, Germany).

### Functional Analysis of *OsNPF7.2* in *Xenopus laevis* Oocytes

The *pT7TS* (Cleaver et al., 1996) was used as the backbone for all the *Xenopu*s oocytes expression vectors. For *pT7TS-OsNPF7.2* and *pT7TS-AtNPF6.3*, the CDS of *OsNPF7.2* or *AtNPF6.3* was inserted separately into the backbone via *Bgl*II and *Spe*I.

The nitrate transport activity of OsNPF7.2 was measured as described previously (Tong et al., 2005) with some modification. Briefly, the CDS of *OsNPF7.2* and *AtNPF6.3* were cloned into the *X. laevis* oocytes expression vector *pT7TS*. Capped mRNA (cRNA) was transcribed *in vitro* using mMESSAGE mMACHINE T7 kits (Ambion, USA) following the manufacturer’s instructions. Fifty nano liters of 1 μg μL^-1^ cRNA was injected in each oocyte and the oocytes were incubated in nitrate-free MBS (modified Barth’s saline) for 2 days before treatment. For uptake, the oocytes were exposed overnight to 10 mM or 200 μM of Na^15^NO_3_ in nitrate-free MBS. For efflux, oocytes were injected with 50 nL of 20 mM Na^15^NO_3_ and further incubated in nitrate-free MBS (pH 7.5) for 8 or 24 h respectively. After treatment the oocytes were all washed 6 times with ${NO}_{3}^{-}$ free MBS and dried for 3 days at 70°C. The content of ^15^N was analyzed using an isotope ratio mass spectrometer coupled with N elemental analyzer (IsoPrime100, Elemental Scientific, USA).

## Results

### *OsNPF7.2* Is Mainly Expressed in Elongation and Maturation Zones of Roots

**FIGURE 1:**
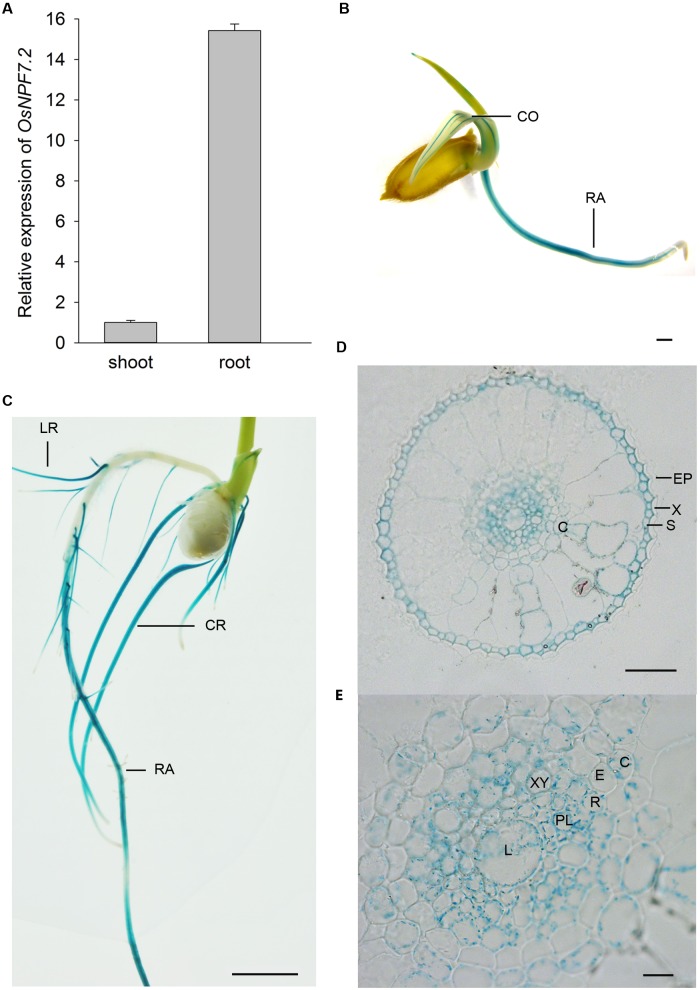
***OsNPF7.2* is mainly expressed in the elongation and maturation zones of roots.** **(A)** qPCR analysis of *OsNPF7.2* expression in 5-day-old rice seedlings. *UBC* was used as reference gene. Data represent mean ± SD from one experiment of six seedlings, three independent experiments showed the same result. **(B–E)** GUS staining of *P_OsNPF7.2_: GUS* seedlings. GUS staining of whole plant **(B)**, roots **(C)**, and cross section of roots **(D,E)**. Bar = 1 mm in **(B)**, 3 mm in **(C)**, 20 μm in **(D)** and 10 μm in **(E)**. CO, coleoptile; RA, radical root; CR, crown root; LR, lateral root; EP, epidermis; X, exodermis; S, sclerenchyma; C, cortex; E, endodermis; R, pericycle; XY, xylem; PL, phloem; L, late metaxylem.

Microarray data analysis showed that *OsNPF7.2* was mainly expressed in the elongation and maturation zones of roots at the vegetative stage as shown in the Rice Expression Profile Database (RiceXPro^2^) (**Supplementary Figures S1A,B**). The qPCR analysis verified that *OsNPF7.2* was mainly expressed in the roots of seedlings (**Figure 1A**). To elucidate a more detailed expression pattern of *OsNPF7.2*, the 1.5-kb upstream region of its CDS was used to drive the expression of *GUS*. The GUS staining analysis further confirmed that *OsNPF7.2* was mainly expressed in the elongation and maturation zones of roots, and in coleoptile of seedlings (**Figures 1B,C**). A weak GUS staining was also detected in the major veins of the leaves (**Supplementary Figure S1C**). The cross sections showed that *OsNPF7.2* was mainly expressed in the root sclerenchyma, cortex and stele (**Figures 1D,E**). The lateral root primordium was not stained (**Supplementary Figure S1D**). The data thus suggests that *OsNPF7.2* mainly functions in the roots.

### High Nitrate Induces *OsNPF7.2* Expression

**FIGURE 2:**
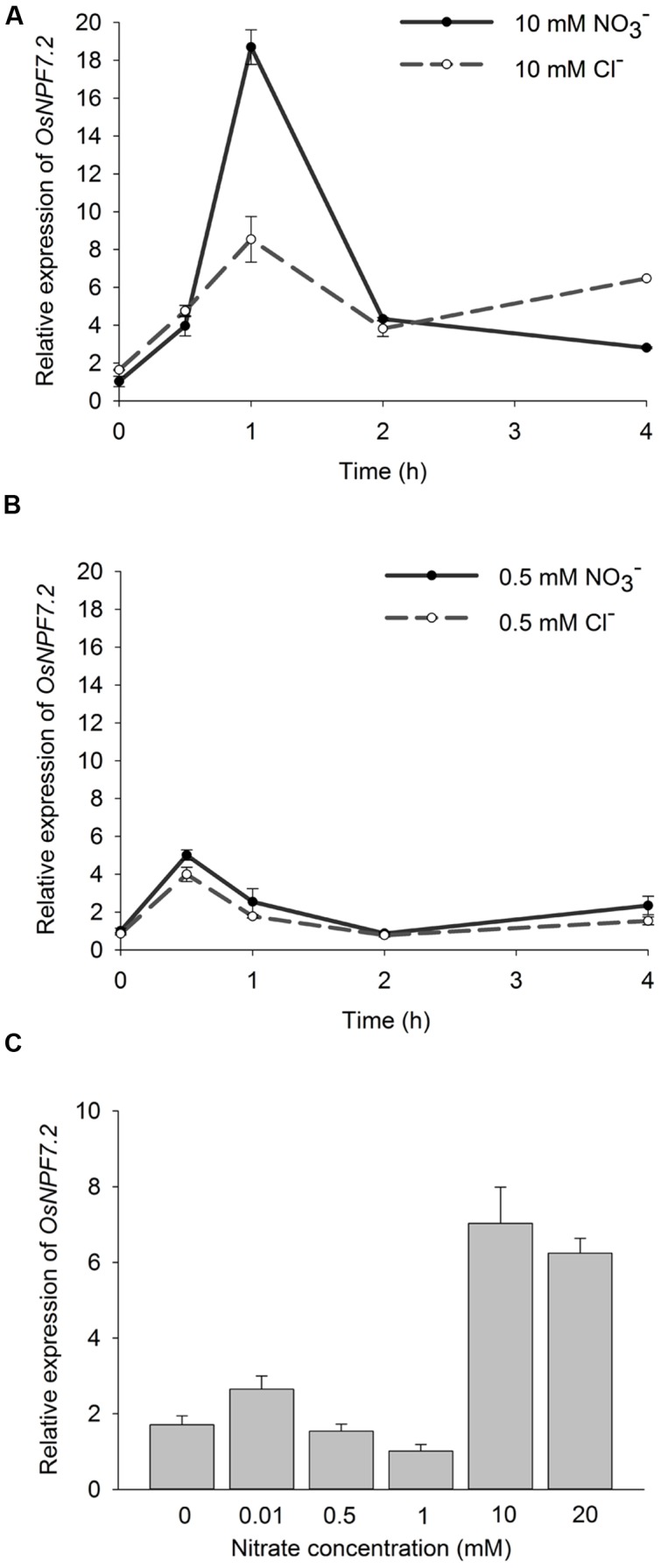
**High nitrate induces *OsNPF7.2* expression in roots.** **(A,B)** qPCR analysis of short-term induction of *OsNPF7.2* in rice roots under 10 mM **(A)** and 0.5 mM **(B)** nitrate solution, respectively. ZH11 seedlings were grown in IRRI solution for 2 weeks, and then transferred for 3-day nitrate starvation, finally shifted in IRRI solution with 10 mM or 0.5 mM KNO_3_ or KCl instead of original N sources. In the solution, equal molar of KCl instead of KNO_3_ was used as control. **(C)** Long-term induction of *OsNPF7.2* in rice roots. ZH11 seedlings were grown on 1/2 MS medium with various concentrations of KNO_3_ for 2 weeks. Ammonium was used to maintain total N concentrations equal in these medium. Data in **(A–C)** represent mean ± SD from one experiment of 10 seedlings, two independent experiments showed same result.

Although a number of *Arabidopsis* NPF members have been demonstrated to transport different types of substrates, including nitrate, amino acids, oligopeptides, phytohormones and glucosinolates, *OsNPF7.2* appears to be the most homologous with two NRTs *AtNPF7.2* (*AtNRT1.8*) (Li et al., 2010) and *AtNPF7.3* (*AtNRT1.5*) (Lin et al., 2008). Therefore, the response of *OsNPF7.2* expression in roots to nitrate was tested using qPCR. In the experiment on shifting from a nitrate starved solution to 10 mM nitrate solution, the *OsNPF7.2* mRNA level in rice roots increased more than 18-fold within 1 h, and subsequently showed a rapid decline. The level in KCl control also increased 5-fold within 1 h (**Figure 2A**). In 0.5 mM nitrate induction, *OsNPF7.2* mRNA level in roots only increased 5-fold within 0.5 h, while that in KCl control also increased 5-fold within 0.5 h (**Figure 2B**). To observe the long-term induction (**Figure 2C**), plants were grown on 1/2 MS medium (Murashige and Skoog, 1962) containing different concentrations of KNO_3_ for 2 weeks. Ammonium was used in the media to maintain the total N concentrations. The expression of *OsNPF7.2* in roots in high concentrations of nitrate (10 and 20 mM) was significantly higher than that in low concentrations of nitrate (0–1 mM) (**Figure 2C**). The data therefore indicates that high concentrations of nitrate induce the expression of *OsNPF7.2*.

### OsNPF7.2 Is Localized on Tonoplast

**FIGURE 3:**
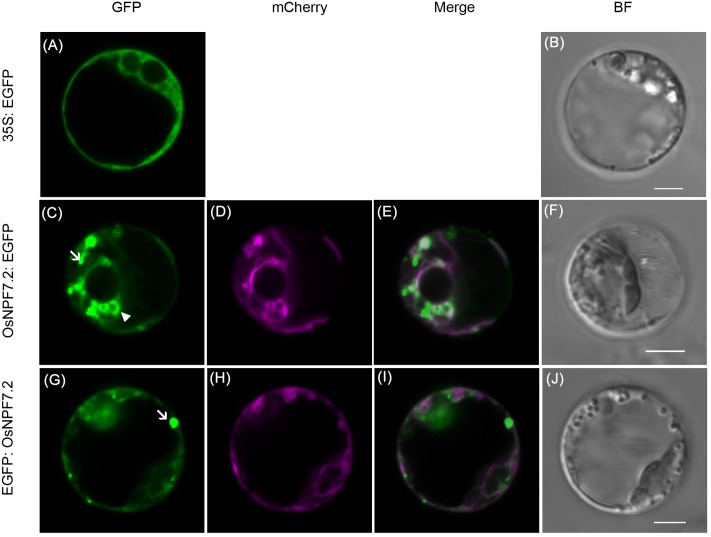
**OsNPF7.2 is mainly localized on tonoplast.** **(A,B)** Rice protoplasts expressing *35S: EGFP* as control. **(C–F)** Rice protoplasts expressing *35S: OsNPF7.2: EGFP* and a tonoplast localized marker *vac-rk*. **(G–J)** Rice protoplasts expressing *35S: EGFP: OsNPF7.2* and a tonoplast localized marker *vac-rk*. BF, bright field; GFP, green fluorescent protein. Merged shows the signal of GFP merged with corresponding mCherry. Arrowhead indicates the small vacuoles can be merged with *vac-rk*. Arrows indicate the small vacuoles cannot be merged with *vac-rk*. Bar = 5 μm.

Members of NPF family have been shown to be localized on the plasma membrane or tonoplast. To determine subcellular localization of OsNPF7.2, the enhanced green fluorescent protein (EGFP) fused to N- or C-terminal of OsNPF7.2 was transiently expressed in rice protoplasts. The EGFP fluorescence signal of *OsNPF7.2: EGFP* (**Figure 3C**) and *EGFP: OsNPF7.2* (**Figure 3G**) partially co-localized with the mCherry fluorescence signal (**Figures 3D,H**) of the tonoplast marker *γ-TIP: mCherry* (*vac-rk*) (Nelson et al., 2007), while free EGFP showed the whole cell fluorescence (**Figures 3A,B**). However, bright small vacuolar structures could also be seen and did not merge with the marker, as the arrows indicated in **Figures 3C,G.** γ-TIP not only marks lytic vacuoles, but also marks protein storage vacuoles and vacuoles storing vegetative storage proteins and pigments (Jauh et al., 1999). Thus the partially localization of OsNPF7.2 with γ-TIP indicates that OsNPF7.2 may be localized not only on the tonoplast of known types of vacuoles, but also to other kinds of vacuole. A partial co-localization of OsNPF7.2 with a rice lytic vacuole membrane protein OsTPKa (Isayenkov et al., 2011) also suggested OsNPF7.2 localized on tonoplast (**Supplementary Figure S2A**). To further confirm the localization of OsNPF7.2 *in situ*, we constructed *35S:OsNPF7.2:EGFP* transgenic rice to observe the localization of OsNPF7.2 in root cells. As shown in **Supplementary Figure S2B**, the fluorescence signal distributed at small vacuolar structures beside a larger vacuole. The fluorescence of free EGFP (*35S: EGFP*) could be seen in the cytoplasm and nucleus, but it did not show obvious fluorescence in the small vacuolar structure. These results imply that OsNPF7.2 is mainly localized on the tonoplast.

### OsNPF7.2 Is a Nitrate Transporter

**FIGURE 4:**
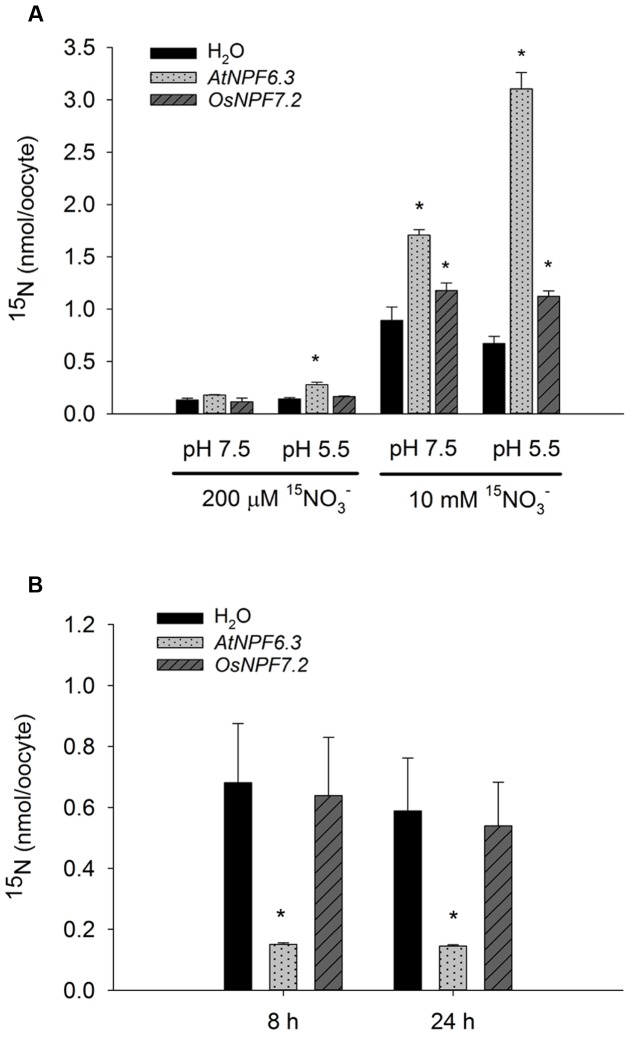
**OsNPF7.2 is a low-affinity NRT.** **(A)** Nitrate uptake assay by *Xenopu*s oocytes expression system. **(B)** Nitrate efflux assay by *Xenopu*s oocytes expression system. *AtNPF6.3* was used as positive control. Water-injected *Xenopu*s oocytes were used as negative control. Data represent means of five oocytes and SD. Two independent experiments showed same result. Asterisks upon the bars indicate statistically significant differences (*P* < 0.05) between the *cRNA*-injected *Xenopu*s oocytes and water-injected *Xenopu*s oocytes by *t*-test.

Four members of the rice NPF family have been demonstrated to be NRTs (Lin et al., 2000; Hu et al., 2015; Li et al., 2015; Xia et al., 2015). We also tested the nitrate transport activity of OsNPF7.2 using the *X. laevis* oocyte expression system. On exposure to 10 mM Na^15^NO_3_ at pH 5.5, the ^15^${NO}_{3}^{-}$ accumulation in *OsNPF7.2*-injected *Xenopu*s oocytes increased by 67% compared with the water-injected *Xenopu*s oocytes (**Figure 4A**). However, at pH 7.5, the ^15^${NO}_{3}^{-}$ accumulation of *OsNPF7.2*-injected *Xenopu*s oocytes increased by 32% compared with the water-injected *Xenopu*s oocytes. This indicates that OsNPF7.2 is a NRT. However, compared to AtNPF6.3, which showed a pronounced difference in ^15^N uptake between pH 5.5 and pH 7.5, nitrate uptake of OsNPF7.2 was less sensitive to the pH change. At the 200 μM Na^15^NO_3_ incubation in pH 5.5, the dual-affinity NRT AtNPF6.3 showed an expected uptake activity, however, OsNPF7.2 did not show any uptake activity. Taking into account that the reported NRTs of NPF are low-affinity NRTs (except AtNPF6.3 and OsNPF6.5), the uptake data in *Xenopu*s oocytes suggests that OsNPF7.2 is a low-affinity NRT.

The sorting signal for the tonoplast of *Arabidopsis* PTRs was reported to be EX_3-5_LL at the N-terminal (Komarova et al., 2012). OsNPF7.2 has the EX_6_LL motif (**Supplementary Figure S3A**). We expected that OsNPF7.2 would be targeted to the plasma membrane, by changing the EX_6_LL motif to EX_6_AA or deleting the LL. Na^15^NO_3_ uptake of the mutated proteins was measured, however, mutations of the putative sorting signal had no effect on the uptake in *Xenopu*s oocytes (**Supplementary Figure S3B**). To investigate whether OsNPF7.2 mediates nitrate efflux, an oocyte efflux measurement was carried out. As shown in **Figure 4B**, the amount of ^15^${NO}_{3}^{-}$ retained in *OsNPF7.2*-injected *Xenopu*s oocytes was almost the same as water-injected *Xenopu*s oocytes, which is unlike *AtNPF6.3* (Leran et al., 2013). The data obtained suggests that OsNPF7.2 is not involved in nitrate efflux in *Xenopu*s oocytes.

### Molecular Analysis of *OsNPF7.2* Knock-Down Mutants

**FIGURE 5:**
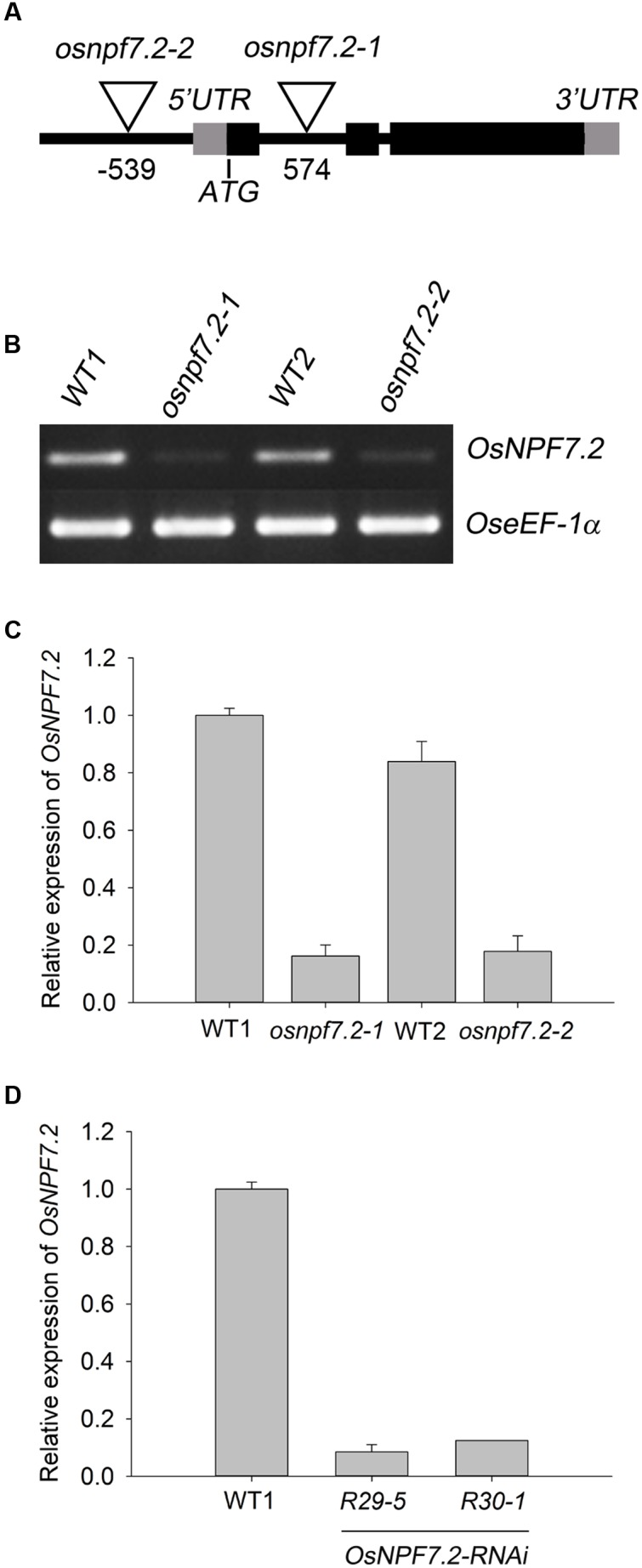
***osnpf7.2* mutants and *OsNPF7.2-RNAi* lines are knock-down lines of *OsNPF7.2*.** **(A)** Diagram of the insertional positions of retrotransposon *Tos17* and T-DNA in two *osnpf7.2* mutants. *osnpf7.2-1* is inserted by *Tos17*, and *osnpf7.2-2* is inserted by T-DNA. **(B,C)** Detection of *OsNPF7.2* expression level in two *osnpf7.2* mutants with semi-quantitative-PCR **(B)** and qPCR **(C)**. **(D)** Detection of *OsNPF7.2* expression level in *OsNPF7.2-RNAi* lines with qPCR. Data of **(C,D)** represent mean ± SD from one experiment of 10 seedlings, three independent experiments showed same result.

To investigate the function of *OsNPF7.2*, two mutants of the gene were obtained from RMD (Rice Mutant Database^3^) and RISD DB (Rice T-DNA Insertion Sequence Database^4^), respectively. The mutant *osnpf7.2-1* was generated from ZH11 (WT1) by a retrotransposon *Tos17* insertion in the first intron of *OsNPF7.2* (**Figure 5A**) (Zhang et al., 2006). The other mutant, *osnpf7.2-2* was generated from *japonica* variety Hwayoung (WT2) by T-DNA insertion in the promoter of *OsNPF7.2* (**Figure 5A**) (Jeong et al., 2006). Flanking sequencing of the PCR fragments verified the insertions in the two mutants. Southern blot analysis showed that *osnpf7.2-2* contained one copy of T-DNA insertion (**Supplementary Figure S4**). The insertion copy number of *Tos17* inserted in *osnpf7.2-1* was not detected, since *Tos17* is multicopy retrotransposon in rice and activated by tissue culture (Hirochika, 2001). Semi-quantitative-PCR and qPCR showed that *OsNPF7.2* expression in the two *osnpf7.2* mutants was decreased, when compared with those of their corresponding wild type (**Figures 5B,C**). The qPCR primers were based on first and second exon to span the insertion site of *osnpf7.2-1. Tos17* was inserted into the intron of *OsNPF7.2-1*, that might influence the splicing efficiency; therefore, a part of mRNA can be spliced successfully. *osnpf7.2-2* was inserted by T-DNA in the promoter of *OsNPF7.2*, that might influence the efficiency of *OsNPF7.2* transcription.

To validate the results from the *osnpf7.2* mutants, the RNA interference plants of *OsNPF7.2* (*OsNPF7.2-RNAi*) were generated in ZH11 (WT1) background. Southern blot analysis showed that line 29-5 and line 30-1 of *OsNPF7.2-RNAi* contained two and one copy(s) of the *OsNPF7.2-RNAi* fragment, respectively (**Supplementary Figure S4**). The qPCR result revealed that expression of *OsNPF7.2* significantly decreased in these two lines (*R29-5* and *R30-1*) (**Figure 5D**). They were subsequently used for further analysis.

### Knock-Down of *OsNPF7.2* Affects Rice Growth under High ${NO}_{3}^{-}$ Condition

**FIGURE 6:**
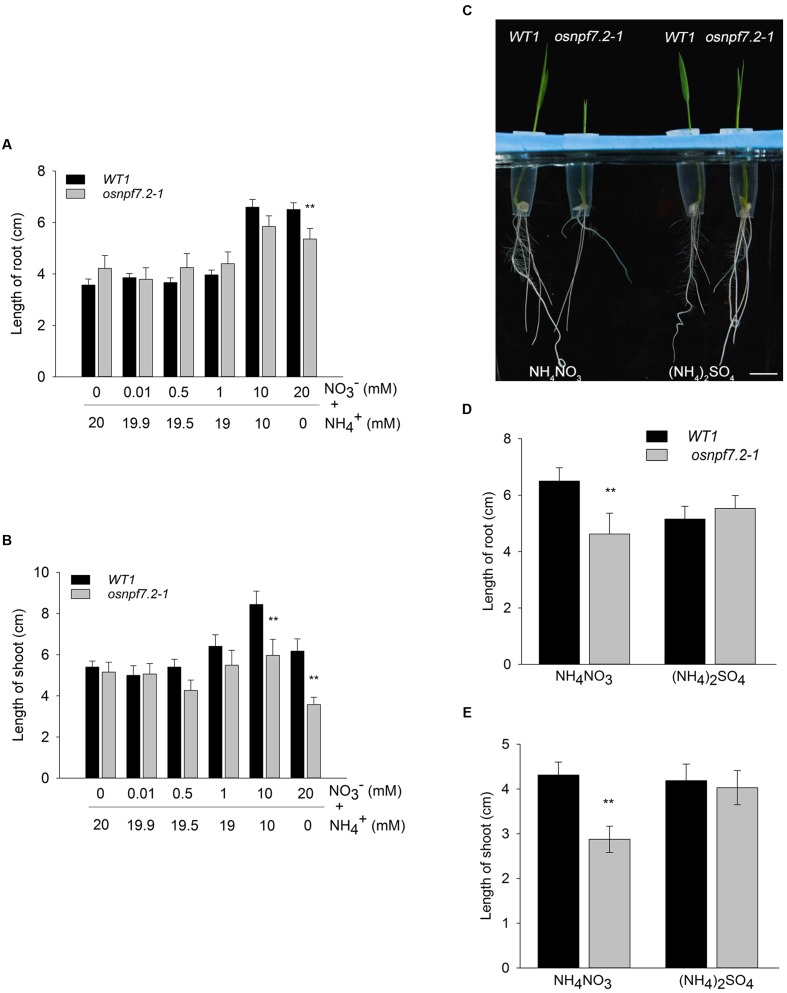
***osnpf7.2-1* shows retardant growth under high concentrations of nitrate.** **(A,B)** Root and shoot length of *osnpf7.2-1* and wild type plants (ZH11). Wild type and mutant plants grew on 1/2 MS medium containing various concentration of KNO_3_ for 7 d. Ammonium was used to maintain the total N concentrations in the medium. **(C–E)** Root and shoot length of *osnpf7.2-1* and wild type plants in 10 mM NH_4_NO_3_ and (NH_4_)_2_SO_4_. Plants were grown on 1/2 MS medium containing NH_4_NO_3_ or (NH_4_)_2_SO_4_ as N source for 7 days. Bar = 1 cm in **(C)**. For all the subfigures, data represent means of 16 samples and SE. Two independent experiments showed same result. Asterisks indicate significant differences (*P* < 0.01) between *osnpf7.2-1* and wild type plants (ZH11) by *t*-test.

Because *OsNPF7.2* was induced by a high nitrate concentration (**Figure 2**), the effect of nitrate on growth of the knock-down mutant *OsNPF7.2-1* was further investigated (**Figure 6**). The plants were grown on 1/2 MS medium containing various concentrations of KNO_3_ for 7 days, and total N concentrations was maintained with ammonium in the medium. The root length of *osnpf7.2-1* decreased 11.3 and 17.6% on 10 mM and 20 mM nitrate medium compared to WT1. The shoot length of *osnpf7.2-1* decreased 29.3 and 42% on 10 mM and 20 mM nitrate medium, respectively. However, there was no statistically significant difference between *osnpf7.2-1* and WT1 under lower nitrate concentrations (**Figures 6A,B**).

To confirm that the difference in growth is caused by nitrate and not by ammonium, the plants were also grown in a medium containing 10 mM NH_4_NO_3_ or (NH_4_)_2_SO_4_ as N source. Length of root and shoot of *osnpf7.2-1* decreased 28.8 and 33.3% than that of WT1, when grown on NH_4_NO_3_ medium, but these differences were not present in plants on (NH_4_)_2_SO_4_ medium (**Figures 6C–E**). This indicates that the growth difference between *osnpf7.2-1* and wild type is caused by nitrate.

**FIGURE 7:**
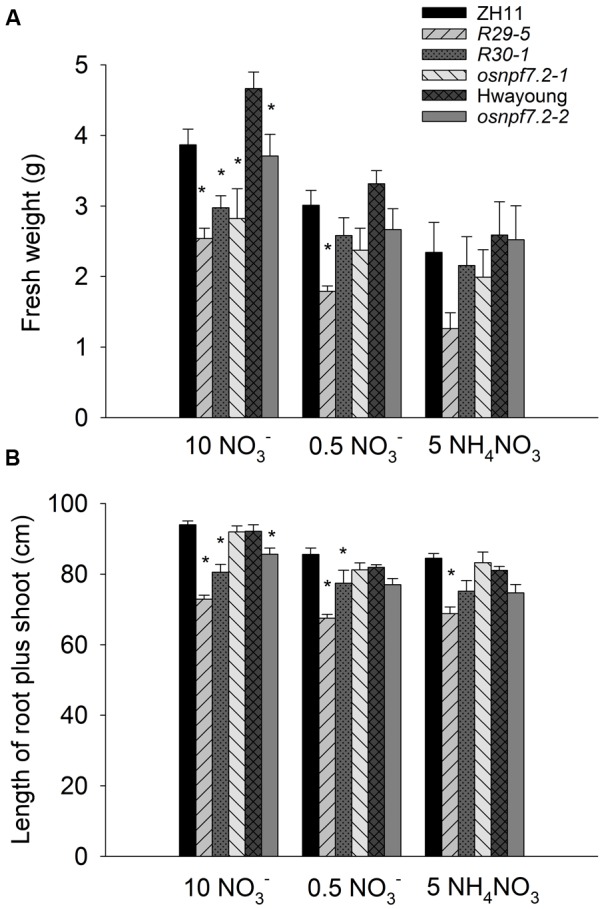
***OsNPF7.2-RNAi* plants show retardant growth compared with the wild type plants in hydroponic solution.** **(A)** Fresh weight per plant. **(B)** Length of the whole plants. Plants grew in IRRI solution for 5 weeks, then grew in IRRI solution containing 5 mM Ca(NO_3_)_2_ (10 ${NO}_{3}^{-}$), 0.25 mM Ca(NO_3_)_2_ (0.5 ${NO}_{3}^{-}$) or 5 mM NH_4_NO_3_ (5 NH_4_NO_3_) as N source for 3 weeks. The wild type of the *RNAi* lines (*R29-5* and *R30-1*) and *osnpf7.2-1* is ZH11. The wild type of *osnpf7.2-2* is Hwayoung. Data represent means of 10 samples and SE. Two independent experiments showed same result. Asterisks upon the bars indicate significant differences (*P* < 0.05) between the transgenic line with their corresponding wild type plants in the LSD-test following one-way ANOVA.

To further confirm the effects of high nitrate on growth of *osnpf7.2* mutants (**Figure 6**), two *OsNPF7.2-RNAi* lines as well as *osnpf7.2* mutants were grown in IRRI solution containing 10 mM nitrate, 0.5 mM nitrate, and 5 mM NH_4_NO_3_ as N source. Similar to the *osnpf7.2* mutants, the *OsNPF7.2-RNAi* plants also showed a decrease in fresh weight, compared to their wild type, under high nitrate supply (**Figure 7**). As shown in **Figure 7A**, in 0.5 mM ${NO}_{3}^{-}$ solution, only *OsNPF7.2-RNAi 29-5* plants showed a decrease in fresh weight, while all the knock-down lines showed decreased fresh weight in 10 mM ${NO}_{3}^{-}$ solution. When grown in 5 mM NH_4_NO_3_, *OsNPF7.2-RNAi 29-5* line showed decreased fresh weight, which did not reach a statistical significance (**Figure 7A**). For the whole plant length, in 0.5 mM ${NO}_{3}^{-}$ solution, only *OsNPF7.2-RNAi* plants showed decreased length, while *OsNPF7.2-RNAi* and *osnpf7.2-2* mutant showed decreased length in 10 mM ${NO}_{3}^{-}$ solution. When grown in 5 mM NH_4_NO_3_, only the *OsNPF7.2-RNAi 29-5* line showed a statistically significant decreased length (**Figure 7B**). Biomass data was consistent with the growth data on 1/2 MS medium (**Figure 6**), though the length did not have obvious differences during the later stage in hydroponic experiments. Above all, the data from *OsNPF7.2-RNAi* further verify that knock-down of *OsNPF7.2* retards rice growth in high nitrate. However, knock-down of *OsNPF7.2* did not change the content of N or nitrate in root and shoot, and the nitrate concentration in xylem sap (**Supplementary Figure S5**), indicating that *OsNPF7.2* is not involved in long-distance allocation of nitrate.

## Discussion

Our data showed that *OsNPF7.2* displayed the capacity for nitrate uptake when expressed in *Xenopu*s oocytes and OsNPF7.2 was mainly localized on the membrane of large and small vacuoles. The expression analysis indicated *OsNPF7.2* was expressed mainly in elongation and maturation zone of roots. The knock-down of *OsNPF7.2* affected rice growth under high concentrations of nitrate; however, altered expression of *OsNPF7.2* did not affect the nitrate content of roots and shoots, or translocation of nitrate from roots to shoots. These results suggest that *OsNPF7.2* may play a role in the temporary storage or usage of nitrate in the tonoplast of the root elongation and maturation zone.

Most of the NPF members were found to be localized on the plasma membrane and only AtNPF8.3 (PTR2/NTR1), AtNPF8.4 (PTR4) and AtNPF8.5 (PTR6) were found on the tonoplast (Weichert et al., 2012). It is proposed that the motif ([D/E]X_3-5_L[L/I]) in the cytosolic N-terminal region is required for tonoplast localization of NPF proteins, and the loop between the transmembrane domain 6 and 7 is required for the plasma membrane localization (Komarova et al., 2012). OsNPF7.2 has an EX_6_LL motif in the N-terminal region (**Supplementary Figure S3A**). The transient expression of *OsNPF7.2* in rice protoplasts showed that it was mainly localized on the large (lytic) vacuolar membrane, and some small vacuolar membrane (**Figure 3**; **Supplementary Figure S2**). Plant cells are considered to possess functionally different types of vacuoles in a same cell. For example, both protein storage and lytic vacuoles (LV) have been characterized at root meristems of barley and pea seedlings (Olbrich et al., 2007). The protein storage vacuoles (PSV) were shown as small vacuoles in root elongating cells (Fluckiger et al., 2003). Another type of vacuole storing vegetative storage proteins (VSPs) was also identified as small vacuoles within the cytoplasm (Jauh et al., 1998). Spherical structures were observed within the lumen of LVs in rapidly expanding young cotyledons cells of *Arabidopsis* (Saito et al., 2002). The structures were undefined and emitted strong fluorescence than LVs in *γ-TIP: GPF* line. γ-TIP marks not only LV, but also PSVs and VSPs (Jauh et al., 1999). Thus the partial co-localization of OsNPF7.2 with γ-TIP indicates that OsNPF7.2 may be localized not only on the known types of vacuolar membrane, but also other types of vacuolar structures.

OsNPF7.2 failed to take up the dipeptide Pro-Leu when expressed in *ptr2* yeast mutant (**Supplementary Figure S6**). Although the substrates for NPF proteins are diverse, many NPFs investigated are able to transport nitrate. The heterogeneous expression in *Xenopu*s oocytes showed that OsNPF7.2 could mediate the uptake of nitrate (**Figure 4**), but not as strongly as AtNPF6.3. Hechenberger et al. (1996) suggested that the heterologous expression of CLC proteins and their electrophysiological detection is mainly limited by their localization to the plasma membrane (Hechenberger et al., 1996). The OsNPF7.2 showed tonoplast localization (**Figure 3**) in rice, this might be the reason that it was not well targeted to the plasma membrane in *Xenopu*s oocytes. However, our attempt to alter its localization for better targeting into the plasma membrane was not successful (**Supplementary Figure S3**). Changing the EX_6_LL motif in the N-terminal region to EX_6_AA or deleting the LL had no effect on the uptake activity in *Xenopu*s oocytes. That indicates that the full-length OsNPF7.2 might be localized on plasma membrane in *Xenopu*s oocytes. Taken together with the lower accumulation of ^15^${NO}_{3}^{-}$ in *Xenopu*s oocytes of the other two rice NPFs (OsNPF6.5 and OsNPF2.2) than negative control, suggests there might be other possible reasons. An alternative explanation for the weak transport activity in *Xenopu*s oocytes might be that the codons of rice are not optimized for *Xenopu*s oocytes (Feng et al., 2013). High GC content of rice *NPF* genes might affect their expression level in *Xenopu*s oocytes. *OsNPF2.4* had been optimized for oocytes and showed a better accumulation of nitrate when expressed in *Xenopu*s oocytes (Xia et al., 2015).

It has been shown that 53% of nitrate absorbed from the rhizosphere could be directed into assimilation and vacuolar storage in the roots, and only 37% was translocated to the shoot (Kronzucker et al., 2000). This shows that most nitrate is assimilated in the root or temporarily stored in root vacuoles. *OsNPF7.2* is mainly expressed in the elongation and maturation zones of the root (**Figure 1**) and encodes a protein that is localized on the tonoplast (**Figure 3**). However, the *osnpf7.2* mutants did not show defective translocation of nitrate to the shoot when compared with the wild type (**Supplementary Figure S5**). This implies that *OsNPF7.2* may play a role in intracellular nitrate homeostasis. The nitrate concentration inside the vacuole of rice can reach up to a 40 mM level (Fan et al., 2007). This suggests that rice plants may require low affinity NRTs for export and import of nitrate to the vacuole. OsNPF7.2 may be involved in such a process. The pH of cytoplasm is about 7.0 to 8.0, OsNPF7.2 did not exhibit a large difference in the nitrate uptake between pH 5.5 and pH 7.5 (**Figure 4A**). This suggests that OsNPF7.2 may be involved in import of nitrate to the tonoplast in plants. Although the concentrations of nitrate in the cytosol in plant are likely to be lower than the 10 mM used in the oocyte experiments (**Figure 4**), they are reported to be in the low-affinity (mM), but not high-affinity (μM) range (Miller and Smith, 1996). In rice, OsNPF7.2 may be functionally orthologous to AtCLCa, a vacuolar NRT in *Arabidopsis* (De Angeli et al., 2006). However, AtCLCa is an antiporter, but NPFs are symporters. This may also suggest that OsNPF7.2 transports nitrate out of vacuoles, considering the more protons in vacuoles. Due to lack of experimental data, it is difficult to conclude whether OsNPF7.2 functions in nitrate storage into the vacuole or remobilization out of the vacuole but in oocytes no efflux activity was detected.

Distinctive from the cell type-specific tissue localization of *Arabidopsis* NPFs, rice NPFs showed a broader localization. For example, transcripts of *AtNPF6.3* accumulated primarily in the epidermal tissue in newly differentiated cells, mostly in the cortex or endodermis of mature parts of the root (Huang et al., 1996). However, rice *OsNPF6.5/OsNRT1.1B* was expressed in a wider range of cell types including root hairs, epidermis and vascular tissues (Hu et al., 2015). Two *Arabidopsis* NPF7 subfamily members *AtNPF7.3* and *AtNPF7.2* were expressed in the root pericycle cells close to the xylem (Lin et al., 2008) and in xylem parenchyma cells (Li et al., 2010), respectively; while rice *OsNPF7.2* was expressed in the cortex and the stele (**Figure 2**). This indicates that the *OsNPF7.2* might play a different role in the rice root from *AtNPF7.3* and *AtNPF7.2*.

Under high nitrate supply, the *OsNPF7.2* knock-down plants showed retarded growth compared with their wild type (**Figure 6**), and high nitrate induced expression of *OsNPF7.2*. Though the control KCl treatment also induced expression of *OsNPF7.2* by 5 fold, the 10 mM KNO_3_ induced the expression of *OsNPF7.2* by 18 fold. This implies that the K or Cl might contribute to the five-fold induction, but does not influence the conclusion that there is nitrate induction of *OsNPF7.2*. However, knock-down of *OsNPF7.2* did not cause a severe phenotype on growth in high nitrate and did not a cause distinctive phenotype in low nitrate except for the RNAi line *R29-5*. The line *R29-5* had two T-DNA inserts, therefore it could not be excluded that the double insertion sites affected its growth. We hypothesize that the weak phenotype of knock-down *OsNPF7.2* plants may be due to two reasons: (1) the expression of *OsNPF7.2* was not entirely suppressed in the knock-down plants (**Figure 5**); (2) there are two closely related homologous genes (*OsNPF7.3* and *OsNPF7.4*) (Léran et al., 2014) of *OsNPF7.2* in rice and there may be some redundancy.

Curiously for a vacuolar transporter the tissue nitrate content and nitrate translocation did not show differences between knock-down *OsNPF7.2* plants and wild type plants (**Supplementary Figure S5**). It could also be that nitrate might not be the sole substrate of OsNPF7.2, which can also transport other metabolites or hormones as suggested by the data in yeast and plants for many other NPF members. Although many members of the NPF showed peptide or nitrate transport activity, some members in this family transport neither peptides nor nitrate. It seems that only a limited number of members transport peptides or nitrate in the *Arabidopsis* NPFs (Leran et al., 2015). The yeast *ptr2* mutant Y06009 had been used to screen 26 NPFs for the dipeptide Leu-Leu transport activity. Only two members (AtNPF8.1 and AtNPF8.3) previously reported as peptide transporters could be screened using this assay (Leran et al., 2015). *Xenopu*s oocytes were also used for the nitrate transport screening. Their results revealed that a few previously investigated NPFs were confirmed to be able to transport nitrate, but at least two proteins that mediated nitrate influx into oocytes reported in previous studies were not confirmed in this screening. They are AtNPF2.13 (NRT1.7) (Fan et al., 2009) and AtNPF4.6 (NRT1.2) (Huang et al., 1999). The NPF family proteins have been showed to transport several different substrates. Some NPF transporters even had the ability to transport both nitrate and hormones (Krouk et al., 2010; Kanno et al., 2012; Léran et al., 2014). However, three homologous genes (*AtNPF7.1. AtNPF7.2*, and *AtNPF7.3*) of *OsNPF7.2* did not show transport activity for ABA, GA, and JA-Ile (Chiba et al., 2015). No other members from subfamily 7 of the *NFP* family have been characterized in other plants. So, more systematic work is required to identify other potential substrates of OsNPF7.2, and transportomics (Krumpochova et al., 2012) may be a very useful solution for this problem in future studies.

## Author Contributions

MZ designed the research. RH, DQ, YC, and XP performed the experiments. RH, and DQ carried out vector construct, transgene plant generation, physiology experiments, subcellular localization, yeast assay and expression analysis. YC and XP designed and carried out *Xenopu*s oocytes uptake measurement. MZ and RH drafted the manuscript. YC, AM, and XF revised the manuscript. All authors approved the manuscript.

## Conflict of Interest Statement

The authors declare that the research was conducted in the absence of any commercial or financial relationships that could be construed as a potential conflict of interest.

## Footnotes

1 http://ricexpro.dna.affrc.go.jp/

2 http://ricexpro.dna.affrc.go.jp/

3 http://rmd.ncpgr.cn/

4 http://cbi.khu.ac.kr/RISD_DB.html

## Abbreviations

CDS: coding DNA sequence
GFP: green fluorescent protein
GUS: β-glucuronidase
MBS: modified Barth’s saline
MCS: multi cloning site
N: nitrogen
NPF: NRT1/PTR family
NRT: nitrate transporter
qPCR: real-time quantitative polymerase chain reaction
UTR: untranslated region
WT1: wild type variety Zhonghua 11
WT2: wild type variety Hwayoung
