# Supplementary material for: Knock-Down of a Tonoplast Localized Low-Affinity Nitrate Transporter OsNPF7.2 Affects Rice Growth under High Nitrate Supply
Source: Front Plant Sci. 2016 Oct 25;7:1529. doi: 10.3389/fpls.2016.01529 (PMC5078692; doi:10.3389/fpls.2016.01529)
Supplement: Supplementary file 2 [file Data_Sheet_1.pdf]

Supplementary Material

Knock-down of a tonoplast localized low-affinity nitrate transporter *OsNPF7.2* affects rice growth under high nitrate supply

Rui Hu[1,2,&], Diyang Qiu[1,2,&], Yi Chen[3], Anthony J Miller[3], Xiaorong Fan[4], Xiaoping Pan[1] and Mingyong Zhang[1*]

*** Correspondence:**
zhangmy@scbg.ac.cn

Supplementary Figure legends

Figure S1

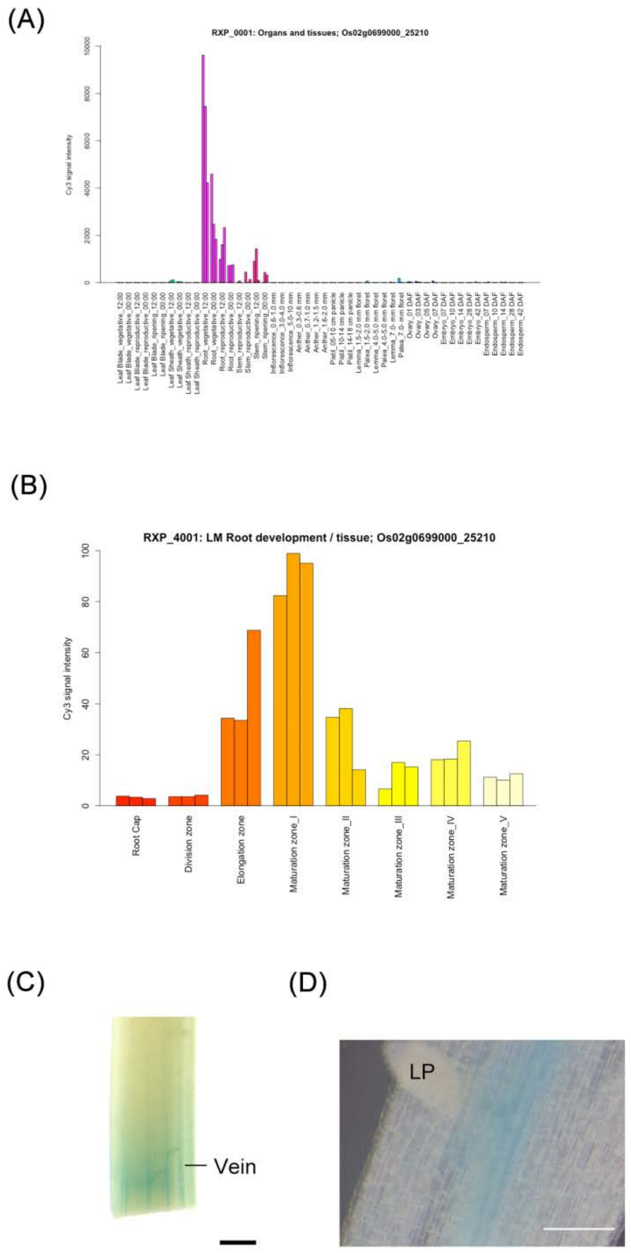

Figure S1. Tissue Expression pattern of *OsNPF7.2.* The gene is mainly expressed in the rice root elongation zone and mature zone. **(A)** Organ and tissue expression data of *OsNPF7.2* from RiceXPro. **(B)** Root development and tissue expression data of *OsNPF7.2* from RiceXPro. **(C)** and **(D)** Gus staining of $P_{OsNPF7.2}$*: GUS* in leaf (C) and lateral root primordium (D). Leaf and root were stained overnight. LP, lateral root primordium. Bar = 1 mm in (C) and 2 mm in (D).

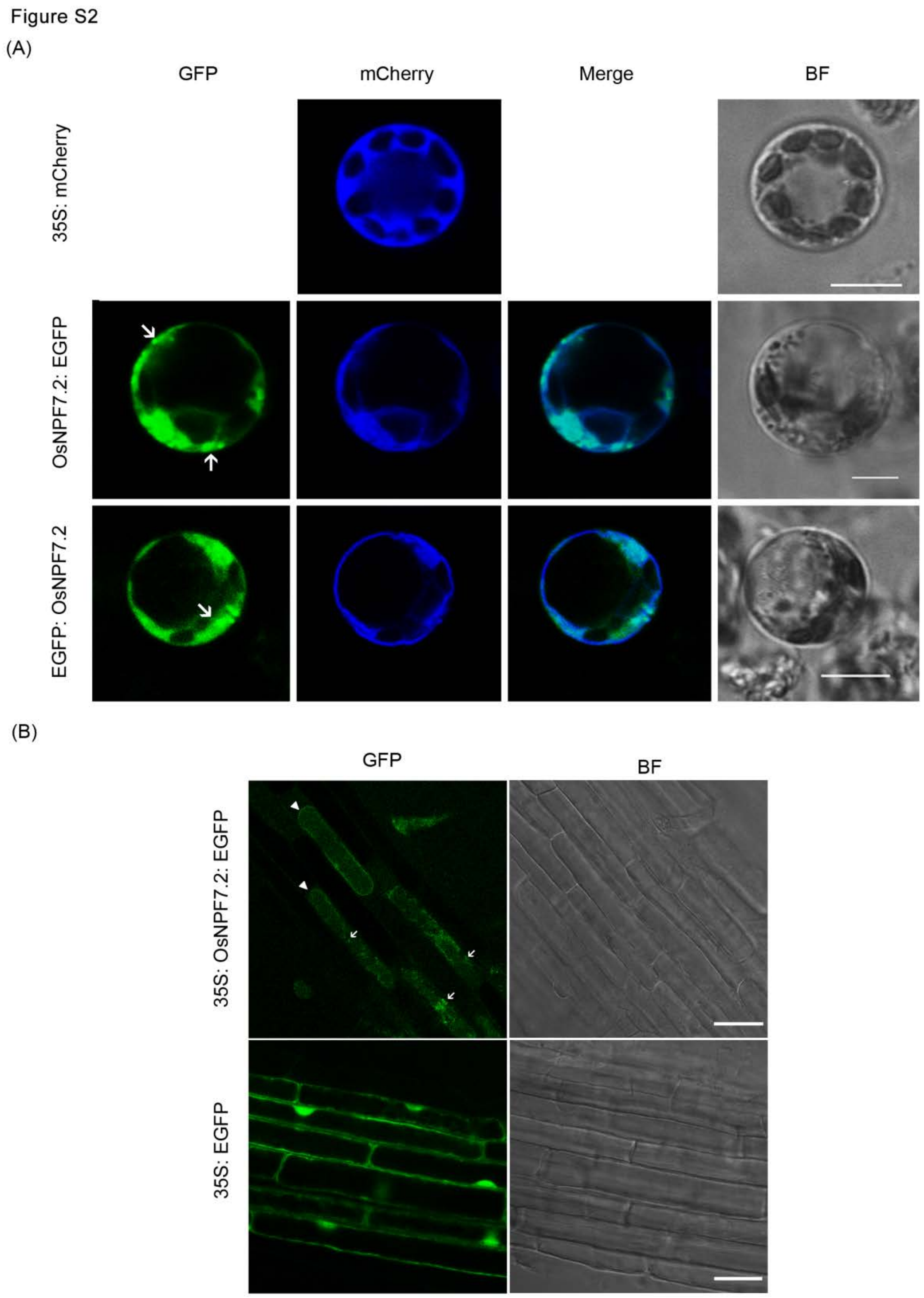

Figure S2. OsNPF7.2 is mainly localized on the tonoplast. (A) Co-localization of *35S: OsNPF7.2: EGFP* with a tonoplast localized marker *35S: OsTPKa: mCherry*. The images were coded green for GFP and blue for mCherry. Arrows indicate the small vacuoles. Scare bars are 7.5 µm, 5 µm, and 7.5 µm from upper to lower panels. TPKa is localized on lytic vacuole membrane of rice aleurone (Isayenkov *et al.*, 2011). For *35S: OsTPKa: mCherry* construction, the *mCherry* was cloned from the *vac-rk* (Nelson *et al.*, 2007) and substituted the *nEYFP* of *pSAT1A-nEYFP-N1* via *Xba*I and

*Kpn*I, then the CDS of *OsTPKa* was fused to *mCherry* via *Xho*I and *Eco*RI. Moreover, a linker $(GGGS)_2$ was inserted between the CDS of *OsTPKa* and *mCherry*. **(B)** Localization of OsNPF7.2 in rice root. Arrowheads indicate the lager vacuole. Arrows indicate the small vacuoles. Scare bars are both 20 µm. *OsNPF7.2: EGFP* fusion protein was drove by *35S* promoter and inserted into *pCAMBIA1301* to generate transgenic plants. Positive control was generated by *35S: EGFP* cloned into *pCAMBIA1301*. Roots of the transgenic plants were observed with confocal laser scanning microscope (Leica TCS SP5, Germany) with 488 nm exciting wavelength for GFP.

Figure S3

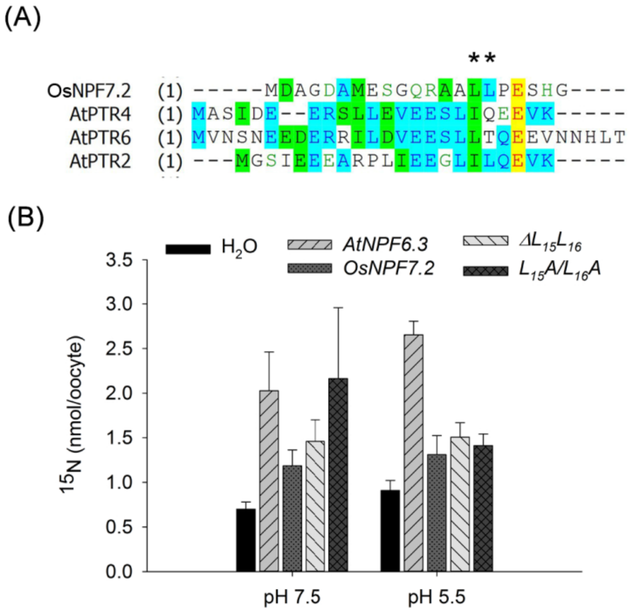

Figure S3. Mutation of the tonoplast-localization motif of OsNPF7.2 has no effect on nitrate uptake in *Xenopus* oocytes. (A) EX_6LL motif on N terminal of OsNPF7.2. Asterisks indicate the $L_{15}L_{16}$ of the motif. **(B)** Nitrate uptake of the $L_{15}L_{16}$ mutation of OsNPF7.2. $\Delta L_{15}L_{16}$ is the construct with a deletion of $L_{15}L_{16}$ of OsNPF7.2. $L_{15}A/L_{16}A$ is the construct mutated L_{15} to A and L_{16} to A of OsNPF7.2. For the mutation $L_{15}A/L_{16}A$ and deletion $\Delta L_{15}L_{16}$ of *OsNPF7.2*, overlapping PCR was used and the mutated sequences were cloned to the *pT7Ts-OsNPF7.2* via the *Hind*III and *Not*I. Data represent mean±SD (n=5).

Figure S4

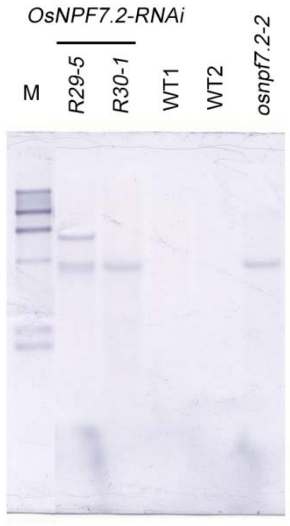

Figure S4. Southern blot analysis of *OsNPF7.2-RNAi* plants and *osnpf7.2-2*. Genomic DNAs were digested with *Hind*III and *BamH*I, and hybridized to a probe of approximately 800-bp sequence from *Hpt* of T-DNA. R, RNAi lines; M, DNA Molecular-Weight Marker II DIG Labeled (Roche, Switzerland).

Figure S5

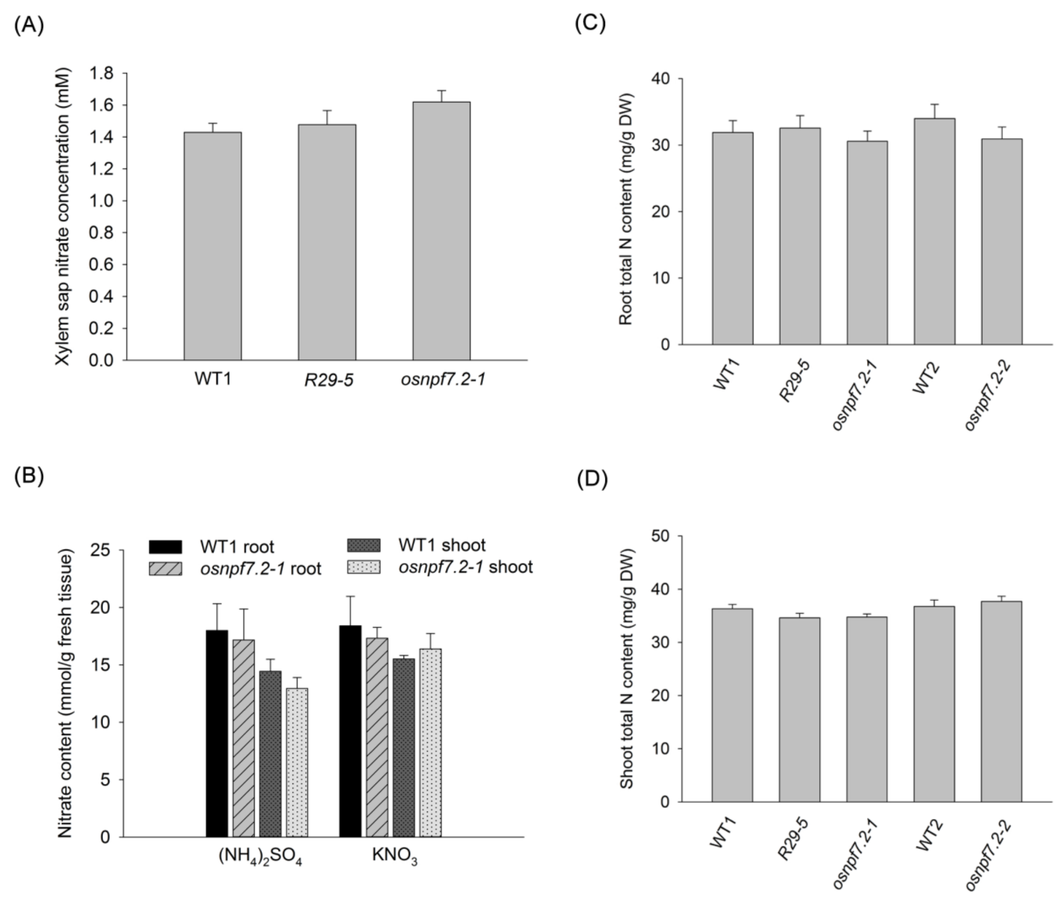

Figure S5. Knock-down plants of *OsNPF7.2* have no differences of xylem sap nitrate concentration, total N content, and tissue nitrate content compared with wild type. (A) Nitrate concentration in xylem sap. Six-week seedlings placed under N-starvation for 3 d, and then transferred to IRRI solution containing 5 mM $Ca(NO_3)_2$. Plants were cut 4 cm above roots immediately and xylem sap collected for 2 h. Data represent mean±SE (n=6). **(B)** Nitrate content in root and shoot. Two-week seedlings were N starved for 4 d, and transferred to IRRI solution containing 20 mM KNO_3 or 10 mM $(NH_4)_2SO_4$ for 24 h, then washed to measure nitrate content by HPLC. Data are mean±SE (n=12) from three replicates. **(C)** and **(D)** Total N content in roots and shoots. Six-week seedlings were transferred to IRRI solution containing 10 mM KNO_3 for 24 h, roots and shoots were separated to measure the total N content. Data represent mean±SE (n=3). Significant differences (P<0.05) between knock-down plants and their corresponding wild-type plants were calculated through LSD-test following one-way ANOVA.

Figure S6

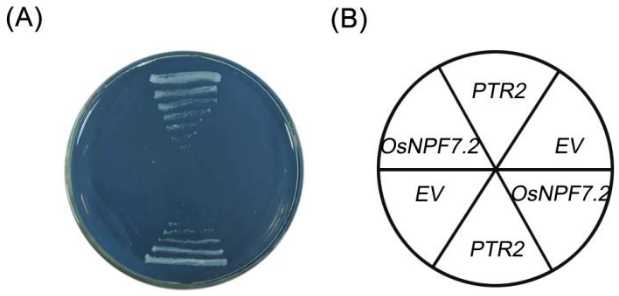

Figure S6. *OsNPF7.2* does not rescue growth of *Saccharomyces cerevisiae ptr2* mutant. Complementation of a *ptr2* mutant strain ABC738 deficient in the uptake of leucine. *EV* is empty vector *pYES260*. *ScPTR2* is positive control from *S. cerevisiae*. The *pYES260* (Melcher, 2000) with modified multi cloning site (MCS) was used as the backbone for all the yeast expression vectors. For *pYES260-OsNPF7.2*, the CDS of *OsNPF7.2* was cloned into *pYES260* via *Hind*III and *Xba*I. For *pYES260-PTR2*, the CDS of *PTR2* from yeast genomic DNA was cloned into *pYES260* via *Bam*HI and *Xba*I. *Saccharomyces cerevisiae* strain ABC738 (*MATa ura3-52 leu2-Δ1 lys2-801 his3-Δ200 trpl-Δ63 ade2-101 ptr2Δ::KanMX2*) was transformed according to Yeastmaker™ Yeast Transformation System 2 User Manual (Clontech, USA), and the transformants were selected on SC-Ura medium. To test peptide transport activity, the transformants were selected on SC-Ura/-Leu medium containing galactose as carbon source and 1mM Pro-Leu as sole source of the required amino acid leucine.

Reference

Isayenkov, S., Isner, J.-C., and Maathuis, F. J. M. (2011). Rice two-pore K^+ channels are expressed in different types of vacuoles. *Plant Cell*. 23, 756-768. DOI: 10.1105/tpc.110.081463

Melcher, K. (2000). A modular set of prokaryotic and eukaryotic expression vectors. *Analytical Biochemistry*. 277, 109-120. DOI: 10.1006/abio.1999.4383

Nelson, B. K., Cai, X., and Nebenfuehr, A. (2007). A multicolored set of in vivo organelle markers for co-localization studies in *Arabidopsis* and other plants. *Plant Journal*. 51, 1126-1136. DOI: 10.1111/j.1365-313X.2007.03212.x
